# Forensic age estimation based on fast spin-echo proton density (FSE PD)–weighted MRI of the distal radial epiphysis

**DOI:** 10.1007/s00414-021-02505-2

**Published:** 2021-01-28

**Authors:** Oguzhan Ekizoglu, Ali Er, Mustafa Bozdag, Negahnaz Moghaddam, Silke Grabherr

**Affiliations:** 1 Department of Forensic Medicine, Tepecik Training and Research Hospital, Güney mahallesi 1140/1 Yenisehir - Konak, Izmir, Turkey; 2 Unit of Forensic Imaging and Anthropology, University Center of Legal Medicine Lausanne-Geneva, Lausanne, Switzerland; 3 Department of Radiology, Tepecik Training and Research Hospital, Izmir, Turkey; 4 Swiss Human Institute of Forensic Taphonomy, University Center of Legal Medicine Lausanne-Geneva, Lausanne, Switzerland; 5 University Center of Legal Medicine Lausanne-Geneva, Lausanne, Switzerland

**Keywords:** Age determination from skeletons, Radius, Forensic medicine, Forensic anthropology, Magnetic resonance imaging

## Abstract

Radiation exposure is a crucial factor to consider in forensic age estimation. The various magnetic resonance imaging (MRI) modalities used in forensic age estimation avoid radiation exposure. This study examined the reliability of distal radius ossification using fast spin-echo proton density (FSE PD)–weighted MRI to estimate age. Left wrist MRI findings of 532 patients aged 10–29 years were evaluated retrospectively using the five-stage system of Dedouit et al. The intra- and interobserver reliability values were *κ* = 0.906 and 0.869, respectively. Based on the results, the respective minimum ages estimated for stages 4 and 5 were 13.4 and 16.1 years for females, and 15.1 and 17.3 years for males; the method could not estimate an age of 18 years in any case. FSE PD MRI analysis of the distal radius epiphysis provides supportive data and can be used when evaluating the distal radius for forensic age estimation.

**Supplementary Information:**

The online version contains supplementary material available at 10.1007/s00414-021-02505-2.

## Introduction

Estimating the age of individuals is important for civil and legal purposes, especially for protecting unaccompanied minors in the context of increasing refugee movements [1]. Although age estimation is most commonly performed for individuals living in developed and border countries experiencing frequent immigration, it is also important in underdeveloped countries due to the lack of birth records and large number of nonhospital deliveries [2]. In most developed countries, the age of criminal liability is between 14 and 21 years [3], and the age thresholds for various civil rights differ widely among countries. Standardization of forensic age estimation procedures is important, in both the civil and criminal context. Age estimation is also important in sports; for fairness and to protect athletes’ health, it is important to ensure that athletes participate in sports competitions in the appropriate age groups [3]. For these purposes, the international, interdisciplinary Study Group on Forensic Age Diagnostics recommends a combination of physical and radiographic examinations of the left hand, a dental examination, and an orthopantomography evaluation. If ossification of the hand is complete, the degree of clavicular ossification should be evaluated by conventional radiography or computed tomography (CT) [4]. The Forensic Anthropology Society of Europe recommends a similar procedure [5]. Because there is no valid medical indication for forensic age estimation, nonionizing methods are being evaluated as a supportive modality for age estimation. Studies have assessed different epiphyseal areas using ultrasound and magnetic resonance imaging (MRI). For example, both modalities have been used to evaluate the development of distal radial epiphysis [6–16]; other studies have assessed the utility of measurements performed in the knee, ankle, elbow, clavicle, proximal humerus, and iliac crest [17–29].

However, fast spin-echo proton density (FSE PD)–weighted MRI has not yet been performed for forensic age estimation using the distal radial epiphysis. Therefore, this study evaluated the value of FSE PD–weighted MRI of the distal radial epiphysis ossification for examining the developmental stages of the distal radius, and its possible utility for assessing forensic age. A FSE PD sequence that can visualize hyaline cartilage was used. The staging system of Dedouit et al. [30] was applied in various epiphyseal regions.

## Material and methods

### Subjects

This study retrospectively examined 620 left wrist MR images of individuals aged 10 to 29 years with known birth dates. The study was approved by the local ethics committee. This type of study does not require formal consent, and all procedures were performed in accordance with the standards of the institutional research committee, and the 1964 Declaration of Helsinki and its later amendments or comparable ethical standards.

MRI was performed in trauma patients between 2018 and 2020 at Tepecik Training and Research Hospital Radiology Clinic. Patients were excluded if they had any wrist pathology or the MRI had motion artifacts. Patients with systemic or neoplastic disorders (potentially affecting bone health and growth, such as an endocrine disease, arthritis, or leukemia), and those on steroid therapy, radiotherapy, or chemotherapy were excluded. The study excluded 88 patients with fractures (47), bone marrow edema (13), surgical fixation (8), hypothyroidism (2), or MRI with motion artifacts (18). Socioeconomic status and ethnicity information were not included in the hospital records. Finally, we evaluated the data of 532 patients (251 males, 281 females; age range: 10–29 years). The hospital information-processing system calculates chronological ages automatically, based on the time between the date of birth (day, month, year) and date of the MRI examination (day, month, year).

### Data acquisition

All examinations involved MRI of the left wrist, performed with a Siemens MAGNETOM Aera 1.5 T machine (Siemens Medical Systems, Erlangen, Germany) with an extremity coil (4-channel flex coil; Siemens). Fat-saturated FSE PD MR images were evaluated in the coronal orientation. The imaging parameters were as follows: TR, 2400 ms; TE, 39 ms; FoV, 140 × 100 mm; voxel size, 0.5 × 0.5 × 3.0 mm^3^; section thickness, 1.5 mm; intersection gap, 10 mm; matrix, 320; number of signal averages, 2; acquisition time, 2.38 s; and reading direction, right to left.

### Image analysis

A Syngo workstation (Siemens Medical Systems) with a high-resolution diagnostic monitor was used to evaluate the MRI scans.

The degree of ossification of the distal radial epiphysis was evaluated as described in Dedouit et al. [30] (Table 1). All slices of the coronal MRI scans were used for staging. Staging decisions were based on the slices showing the most well-developed epiphysis.

**Table 1:** Staging system described by Dedouit et al. [30]

Stage	Description
Stage 1	A continuous horizontal cartilage layer thicker than 1.5 mm was apparent between the junctions of the metaphysis and the epiphysis, and the cartilage was multilaminar in appearance (Fig. 1, S1). The multilaminar appearance was observed as decreased signal intensity in the upper layer, increased signal intensity in the middle layer, and decreased signal intensity in the lower layer.
Stage 2	A continuous horizontal linear cartilage signal intensity was present between the metaphysis and the epiphysis, with a thickness greater than 1.5 mm, with increased signal intensity but without a multilaminar appearance (Fig. 1, S2).
Stage 3	A continuous horizontal linear cartilage signal intensity was present between the metaphysis and the epiphysis, with a thickness less than 1.5 mm and increased signal intensity (Fig. 1, S3).
Stage 4	Discontinuous horizontal linear cartilage signal intensity was present between the metaphysis and the epiphysis, with a thickness less than 1.5 mm and discontinuously increased signal intensity (Fig. 1, S4).
Stage 5	Signal intensity between the metaphysis and the epiphysis was not increased (Fig. 1, S5).

The entire image dataset was evaluated by two experienced observers blinded to patient sex and age. The dataset was assessed twice by the first researcher (radiologist) and once by the second researcher (forensic medicine specialist). The observers, blinded to the previous staging results, reevaluated all images after 4 weeks.

### Statistical analysis

SPSS software (ver. 17.0; SPSS Inc., Chicago, IL, USA) was used for the statistical analysis. The distribution of the defined phases within the cohort was evaluated with descriptive statistics, including minimum age (min), maximum age (max), mean, median, standard deviation (SD), and lower and upper quartiles. The relationship between age and ossification stage was evaluated using Spearman’s correlation rank analysis. Differences between the sexes were analyzed using the Mann–Whitney *U* test. *p* < 0.05 was considered statistically significant. Cohen’s kappa (*κ*) coefficients were calculated to assess the intra- and interobserver reliability [31].

## Results

All staging was based on MRI of the distal radial epiphysis. The mean age of the males and females was 19.4 ± 4.3 and 19.1 ± 4.3 years, respectively (Table 2). The intra- and interobserver evaluations showed good reliability, with *κ* values of 0.906 and 0.869, respectively. Table 3 summarizes the descriptive statistics.

**Table 2:** Age distribution of male and female subjects

Age (years)	Male (*N*)	Female (*N*)
10	10	5
11	9	9
12	8	14
13	8	19
14	9	9
15	18	15
16	16	34
17	22	20
18	23	20
19	13	15
20	15	20
21	13	20
22	23	22
23	20	15
24	12	15
25	8	11
26	10	8
27	7	4
28	2	3
29	5	3
Total	251	281

**Table 3:** Minimum and maximum ages. With means ± SDs. Lower and upper quartiles and medians. At all stages of distal radial epiphysis

Stage	Sex	*N*	Mean ± SD	Min–max	LQ;UQ;median
1	Female	12	11.91 ± 1.23	10.2–13.9	10.77;12.66;12.08
	Male	18	13.10 ± 1.90	10.0–16.0	11.20;14.43;13.50
2	Female	20	12.29 ± 1.10	10.1–13.9	11.41;13.47;12.08
	Male	30	12.67 ± 1.79	10.0–15.9	11.06;14.18;12.29
3	Female	23	14.02 ± 1.36	12.1–16.7	13.00;15.16;13.91
	Male	31	16.39 ± 1.57	12.8–19.8	15.33;17.50;16.41
4	Female	35	15.93 ± 1.25	13.4–18.3	15.25;16.66;15.91
	Male	31	17.71 ± 1.78	15.1–22.6	16.41;19.00;17.25
5	Female	191	21.40 ± 3.30	16.1-29.6	18.66;23.75;21.33
	Male	141	22.72 ± 3.04	17.3-29.6	20.08;24.75;22.58

There were significant sex differences in the proportions of stages 3, 4, and 5 cases (all *p* < 0.01). Spearman’s rank correlation analysis indicated a significant positive relationship between age and stage of ossification of the distal radial epiphysis (all subjects: ρ = 0.809, *p* < 0.01; males: ρ = 0.853, *p* = 0.001; females: ρ = 0.796, *p* < 0.01).

## Discussion

**Fig. 1:**
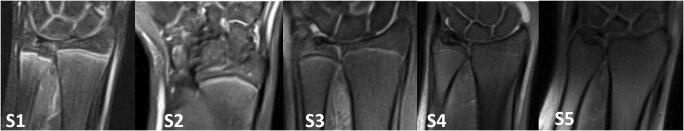
Fast spin-echo proton density (FSE PD)–weighted sequences in coronal orientation on hand wrist MRI: stage 1 to stage 5 for distal radius epiphysis (S1–S5)

This is the first FSE PD MRI study of the ossification stages of the distal radius epiphysis (Fig. 1). Using the staging system of Dedouit et al. [30] for age estimation, the minimum respective ages for stages 4 and 5 were 13.4 and 16.1 years for females, and 15.1 and 17.3 years for males.

Because this is the study to apply FSE PD MRI for evaluating the distal radius epiphysis, the intra- and interobserver agreement data are particularly important. Both were high, likely because the center where the study was conducted, and the observers who analyzed the images, was experienced in the use of MRI for forensic age estimation. As Wittschieber et al. [32] emphasized, observer experience can affect forensic age estimation; therefore, the repeatability of the technique should be assessed in future studies.

Increasing numbers of studies have used nonionizing methods for forensic age estimation. MRI is at the forefront of creating standalone or combined staging systems of the epiphyseal area, using a variety of imaging sequences for forensic age estimation. For instance, T1-SE, T1-TSE, and T1-VIBE sequences have been applied for evaluating the ossified epiphyseal layer of the distal radius [7–16]. Although staging methodologies differ among studies, the Schmeling and Kellinghaus staging system [33, 34], and modified versions thereof [7, 13, 14, 16], are generally used.

FSE PD–weighted MRI can obtain detailed images of epiphyseal cartilage, as described by Dedouit et al. [30]. FSE PD MRI of the knee was used to establish a staging system for age estimation. The Dedouit staging system is based on hyaline cartilage development, and its accuracy and utility have been demonstrated in the context of FSE PD and T2-weighted MRI of the proximal tibia, distal femur, and proximal humerus epiphyses [23, 25, 30]. This methodology provided different minimum age limits to those of T1-weighted MRI studies, for the same epiphyseal areas [24, 26–28, 35]. Examination of the knee and proximal humeral epiphysis with T1-weighted MRI yielded minimum age limits of 14–17 and 14–18 years, respectively, while for both epiphyseal areas the limit was 14–21 years with FSE PD–weighted MRI [23, 30]. However, there have been no age estimation studies based on FSE PD MRI evaluation of the distal radius epiphysis. This suggests that testing this sequence in the context of the distal radial epiphysis may be useful for obtaining other minimum age limits.

This retrospective study had some limitations. Minimum age thresholds can vary, especially with an unbalanced age distribution. Prospective studies with balanced age distributions, along with clinical and socioeconomic evaluations, may be more appropriate for forensic age estimation. However, there are difficulties associated with prospective studies. The reliability of the methods will increase over time, and large retrospective cohort studies of different populations could be performed.

Comparative analysis of our results was impossible because no previous studies that evaluated the distal radial epiphysis by MRI used FSE PD and the Dedouit staging system. Evaluation of the distal radius by MRI is a relatively novel approach, and it may be useful to consider the main results of past studies. Overall, the ages reported in studies of athletes are not comparable due to possible misrepresentation and the influence of developmental characteristics associated with high sports activity. It is also important to determine the minimum ages that can be revealed by population-based forensic age studies. Using T1-weighted MRI, Timme et al. [16] and Er et al. [7] determined minimum age thresholds ranging from 14 to 18 years. In those two studies, the minimum age limits identified using the Schmeling and Kellinghaus staging systems [33, 34], of 17 years for stage 4 and 4a males and 18 years for stage 4b males, are remarkable. Using T1-SE MRI for evaluating stage 3 cases, Serin et al. [15] defined the distal radius epiphysis as being closed in the “triple staging system”; the lowest observed age was 15 years for females and 16 years for males. Moreover, De Tobel et al. [14] used T1-weighted spin-echo images (SE) without fat suppression, and T1-weighted gradient-echo (VIBE) images with fat suppression, in a multiparameter age estimation study. Despite detailed analyses of the distal radius epiphysis, it is impossible to make a valid comparison with recent T1 MRI studies, as De Tobel et al. [14] did not present the minimum age results. Tomei et al. [11] and Serinelli et al. [12] examined the distal radius epiphysis via T1-weighted MRI for age estimation, and found a strong correlation between the results of their combined TW and GP atlas–based method and chronological and estimated ages. However, these studies also failed to present minimum age limits, and did not provide data specific to the distal radius epiphysis.

Our study was retrospective, and it was not possible to obtain socioeconomic or ethnicity data for the study population. Furthermore, the difference between ethnic origins is not an effective factor, while low socioeconomic status may be associated with delayed ossification [36, 37]. The degree to which the study results were affected by socioeconomic status was therefore not determined.

One factor that may affect MRI analyses is the strength of the magnetic field. In their study involving T1-weighted MRI of the distal femoral epiphysis for forensic age estimation, Saint-Martin et al. [35] reported that the magnetic field had no effect. No study has assessed the effects of the magnetic field on FSE PD MRI; comparative studies are therefore needed.

Artifacts can occur in MR images for various reasons, such as hardware factors, texture characteristics, data acquisition and image reconstruction methods, and scanning parameters [38–40]. Methods for correcting many clinical parameters have been developed. However, it is necessary to carefully consider all possible factors affecting new parameters [40]. The current study involved retrospective examination of MRI images, obtained using predetermined technical parameters, to evaluate wrist pathology. FSE PD MRI can image cartilage tissue pathologies [41], and has been used for epiphyseal cartilage imaging for forensic age estimation [23, 30]. However, one of the main limitations of the Dedouit method is the difficulty of determining whether the cartilage thickness is 1.5 mm. Although the intra- and interobserver error was low in this and previous studies [23, 25, 30], this can create difficulties for the observer. It may also be impossible to determine how much of the hyperintense area is affected by possible artifacts. Although no artifacts were reported after excluding 18 cases with motion artifacts in this study, the possible influence of sequence design, coils, and gradients could not be tested due to the retrospective nature of our study.

Additional studies of various sequences, scanning procedures, and equipment are necessary to reduce the disruptive effects of artifacts and improve image quality [38–40]. Chemical shift artifacts, which can affect assessment of hyperintensity in the epiphyseal area, can be reduced by changing the direction of the frequency and phase coding gradients, or by using fat suppression or increasing bandwidth [38].

Future prospective studies may reveal the possible causes of, and means for correcting, artifacts.

Forensic age estimation in living individuals is possible with FSE PD MRI of the distal radius epiphysis, and can provide supportive data for males aged 15–17 years and females aged 14–16 years. FSE PD MR of the distal radius epiphysis, in conjunction with the Dedouit method, shows promise. However, considerable experience may be required for this approach, and it has the disadvantage of requiring a horizontal cartilage thickness measurement.

MRI is potential applicability for age estimation, and eliminates ionizing radiation to ensure patient safety. Future research should investigate this approach in the context of different staging methods, and examine the influence of socioeconomic status and observer experience.

## Supplementary Information

ESM 1 (SAV 6 kb)
